# MP-Align: alignment of metabolic pathways

**DOI:** 10.1186/1752-0509-8-58

**Published:** 2014-05-20

**Authors:** Ricardo Alberich, Mercè Llabrés, David Sánchez, Marta Simeoni, Marc Tuduri

**Affiliations:** 1 Department of Mathematics and Computer Science, University of the Balearic Islands, Ctra. Valldemossa km. 7.5, E-07122 Mallorca, Spain; 2 Department of Environmental Science, Computer Science and Statistics, Ca’ Foscari University of Venice, Dorsoduro 3246 - 30123, Venice, Italy

**Keywords:** Metabolic pathways, Metabolic pathways alignment, Metabolic pathways comparison

## Abstract

**Background:**

Comparing the metabolic pathways of different species is useful for understanding metabolic functions and can help in studying diseases and engineering drugs. Several comparison techniques for metabolic pathways have been introduced in the literature as a first attempt in this direction. The approaches are based on some simplified representation of metabolic pathways and on a related definition of a similarity score (or distance measure) between two pathways. More recent comparative research focuses on alignment techniques that can identify similar parts between pathways.

**Results:**

We propose a methodology for the pairwise comparison and alignment of metabolic pathways that aims at providing the largest conserved substructure of the pathways under consideration. The proposed methodology has been implemented in a tool called MP-Align, which has been used to perform several validation tests. The results showed that our similarity score makes it possible to discriminate between different domains and to reconstruct a meaningful phylogeny from metabolic data. The results further demonstrate that our alignment algorithm correctly identifies subpathways sharing a common biological function.

**Conclusion:**

The results of the validation tests performed with MP-Align are encouraging. A comparison with another proposal in the literature showed that our alignment algorithm is particularly well-suited to finding the largest conserved subpathway of the pathways under examination.

## Background

Metabolism is the chemical system that generates the essential components for life. All living (micro)organisms possess an intricate network of metabolic routes for the biosynthesis of amino acids, nucleic acids, lipids and carbohydrates and for the catabolism of different compounds driving cellular processes. Subsystems of metabolism dealing with specific functions are called metabolic pathways. Over the last ten years these pathways have been the subject of a great deal of research, conducted primarily through two kinds of studies: one focusing on the analysis of single pathways, the other on the comparative analysis of a set of pathways.

The studies that compare metabolic pathways of different species can provide interesting information on their evolution and may help in understanding metabolic functions, which are important in studying diseases and identifying pharmacological targets. In the literature many techniques have been proposed for comparing metabolic pathways of different organisms. Each approach chooses a representation of metabolic pathways that models the information of interest, proposes a similarity or a distance measure and possibly supplies a tool for performing the comparison. The automatization of the whole process is enabled by the knowledge stored in metabolic databases such as KEGG [1], BioModels [2] or MetaCyc [3].

More recent comparative research has proceeded by focusing on alignment techniques that can identify similar parts between pathways, providing further insight for drug target identification [4,5], meaningful reconstruction of phylogenetic trees [6,7], and identification of enzymes clusters and missing enzymes [8,9]. Here too approaches in the literature vary: some consider multiple pathways and identify their frequent or conserved subgraphs [10,11]; others also build their alignments [12-21].

We propose a methodology for the pairwise comparison and alignment of metabolic pathways that aims at providing the largest conserved substructure of the pathways under consideration. The methodology relies on a hypergraph representation of metabolic pathways and defines a reaction similarity score that takes into account the chemical similarity and homology between pairs of reactions. The alignment technique uses the reaction similarity score and the pathway topology to identify the largest conserved subpathway between the two given pathways. The proposed methodology has been implemented in a tool called **MP-Align**, which has been used to perform several validation tests reported herein.

## Methods

This section describes the methodology proposed for the pairwise comparison and alignment of metabolic pathways. We represent metabolic pathways as directed hypergraphs and define a reaction similarity score based on both compound and enzyme similarities. On the basis of these choices we define the alignment algorithm, which has been implemented in **MP-Align**.

### Hypergraph representation of a metabolic pathway

A *directed hypergraph* is a mathematical structure *H*=(*V*,*E*) where *V* is a finite set of nodes and *E* is a set of *directed hyperedges*. A directed hyperedge is an ordered pair of subsets of nodes *E*=(*X*,*Y*); *X* is the set of input nodes of *E* while *Y* is its set of output nodes.

Metabolic pathways can be easily represented as directed hypergraphs: metabolites, enzymes and compounds can be modeled as nodes and reactions as hyperedges. Despite the simplicity of this representation, we made the modeling choices described below. 

− We do not represent ubiquitous substances, such as *H*_2_*O*, phosphate, ADP and ATP as hypergraph nodes. The same is true for enzymes, which are represented as reaction attributes and used to compute the reaction similarity.

− Most of the reactions in metabolic pathways are reversible. A reversible reaction can occur in two directions, from the reactants to the products (forward reaction) or vice versa (backward reaction). The direction depends on the kind of reaction, on the concentration of the metabolites, and on conditions such as temperature and pressure. We model reversible reactions by two corresponding hyperedges, one for the forward reaction and the other for the backward reaction.

− In a metabolic pathway one can distinguish between internal and external metabolites. The former are entirely produced and consumed in the network; the latter represent sources or sinks, that is, connection points produced or consumed by other pathways. We represent external metabolites as input only (*source*) or output only (*sinks*) nodes.

**Figure 1:**
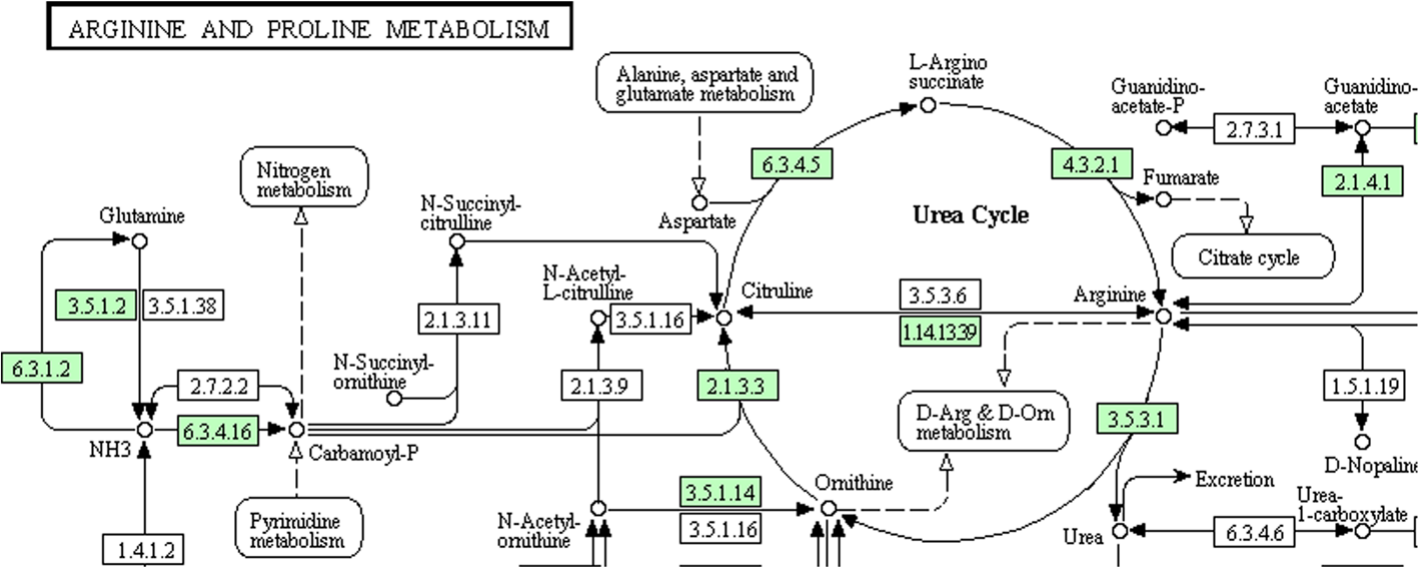
**Part of the KEGG pathway *Arginine and proline metabolism *for *H. Sapiens*.** This figure shows the compounds and enzymes directly involved in the *Urea cycle*.

**Figure 2:**
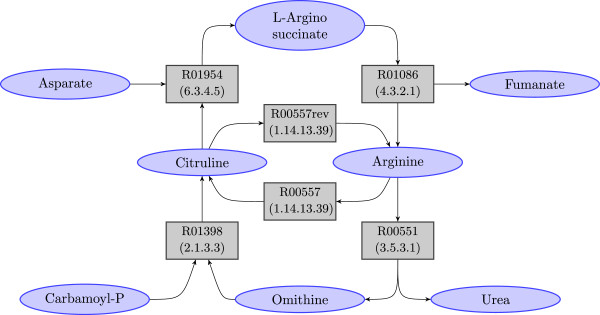
**Hypergraph representation of the *Urea Cycle *shown in Figure **1. Purple nodes represent compounds and grey nodes are hyperedges representing reactions. They specify the catalyzing enzyme as an attribute. For each reaction, the incoming arrows represent the input compounds and the outgoing arrows represent the output compounds. Note that a reversible reaction (e.g. reaction *R00557*) is represented by a forward reaction (grey node with label *R00557*) and a backward one (grey node with label *R00557rev*).

Figures 1 and 2 show a metabolic pathway and its corresponding hypergraph representation. More specifically, Figure 1 shows part of the KEGG *Arginine and proline metabolism* pathway for *H. Sapiens*, focusing on the compounds and enzymes directly involved in the *Urea Cycle*; Figure 2 depicts the hypergraph representation of the cycle itself. Purple nodes in the picture represent compounds and grey nodes are hyperedges representing reactions. Each hyperedge reports both the reaction name (in KEGG nomenclature) and the EC number [22] of the catalyzing enzyme. For each hyperedge, the incoming arrows represent the input compounds of the corresponding reaction and the outgoing arrows represent the output compounds. Note that the reversible reaction *R00557* is translated into two corresponding hyperedges, one for the forward reaction and the other for the backward reaction, which can be distinguished by the suffix ‘*rev*’.

### Reaction similarity score

In the literature there are several approaches to defining a reaction similarity score. Some represent each reaction through the enzyme that catalyzes it and define a score based on enzyme similarity, e.g. [7,19,23]. Other more recent proposals, e.g. [17,18], consider both compound and enzyme similarities. We employ the reaction similarity score defined in [18]. More precisely, let *R*_*i*_=(*I*_*i*_,*E*_*i*_,*O*_*i*_) denote a hyperedge representing a reaction, where *I*_*i*_ is the set of its input nodes (substrates), *E*_*i*_ the enzyme that catalyzes the reaction and *O*_*i*_ the set of its output nodes (products). The similarity score for every pair of reactions *R*_*i*_=(*I*_*i*_,*E*_*i*_,*O*_*i*_) and *R*_*j*_=(*I*_*j*_,*E*_*j*_,*O*_*j*_) is given by the following formula [18]:

(1) $$\begin{array}{l} \mathrm{SimReact}(R_{i},R_{j})=\;\mathrm{SimEnz}(E_{i},E_{j})\cdot w_{e}\\ \;+\mathrm{SimComp}(I_{i},I_{j})\cdot w_{i}\\ \;+\mathrm{SimComp}(O_{i},O_{j})\cdot w_{o} \end{array}$$

where *SimEnz*(*E*_*i*_,*E*_*j*_) is the enzyme similarity between *E*_*i*_ and *E*_*j*_, *SimComp*(*I*_*i*_,*I*_*j*_) is the compound similarity between the input node sets *I*_*i*_,*I*_*j*_ and *SimComp*(*O*_*i*_,*O*_*j*_) is the compound similarity between the output node sets *O*_*i*_,*O*_*j*_. The parameters *w*_*e*_, *w*_*i*_ and *w*_*o*_ are fixed to *w*_*e*_=0.4 and *w*_*i*_=*w*_*o*_=0.3 since, as stated in [18], they provide a good balance between enzymes and compounds.

For the enzyme and compound similarities in (1) we made the following choices. 

− For enzymes, we use the EC hierarchical similarity measure that is based on the comparison of the unique *EC number* (Enzyme Commission number) associated to each enzyme, which represents its catalytic activity. The EC number is a 4-level hierarchical scheme, *d*_1_.*d*_2_.*d*_3_.*d*_4_, developed by the International Union of Biochemistry and Molecular Biology (IUBMB) [22]. Enzymes with similar EC classifications are functional homologues but do not necessarily have similar amino acid sequences. Given two enzymes *e*=*d*_1_.*d*_2_.*d*_3_.*d*_4_ and $e^{\prime }=d_{1}^{\prime }.d_{2}^{\prime }.d_{3}^{\prime }.d_{4}^{\prime }$, their similarity *S*(*e*,*e*^′^) depends on the length of the common prefix of their EC numbers:

$$S(e,e^{\prime })=\max\{i=1,2,3,4:d_{j}=d_{j^{\prime }},\;j=1,\ldots ,i\}/4$$

For instance, the similarity between *arginase* (*e*=3.5.3.1) and *creatinase* (*e*^′^=3.5.3.3) is 0.75.

− For compounds, we use a similarity based on the similarity measure computed by the SIMCOMP (SIMilar COMPound) [24] tool. Given two compounds, the tool represents their chemical structure as graphs and outputs a measure of their maximal common substructure.

Since a reaction may have more than one input (output) compound, we need a way to combine the similarity between pairs of compounds computed by SIMCOMP. Given two sets *X* and *Y* of compounds, the score *SimComp*(*X*,*Y*) is computed by: 

− defining a complete bipartite graph in which the compounds in *X* and *Y* are nodes and the weight of each edge (*x*,*y*)∈*X*×*Y* is the similarity value of *x* and *y* computed by SIMCOMP;

− applying the maximum weighted bipartite matching algorithm to the resulting graph to obtain the best match between *X* and *Y*;

− summing the scores of the best match and dividing it by max{|*X*|,|*Y*|}.

### The **MP-Align** alignment algorithm

This section illustrates the **MP-Align** alignment algorithm. The algorithm receives as input two directed hypergraphs *H*_1_=(*V*_1_,*E*_1_) and *H*_2_=(*V*_2_,*E*_2_) representing two metabolic pathways and gives their similarity score and alignment as output. **MP-Align** has been implemented in Python. The tool is freely available at http://bioinfo.uib.es/~recerca/MPAlign.

The main steps of **MP-Align** follow.

#### Reaction path computation

The first step of the alignment algorithm represents *H*_1_ and *H*_2_ as suitable paths of reactions called *reaction paths*. Given a directed hypergraph *H* representing a metabolic pathway, a *reaction path* is a sequence of reactions (hyperedges) *p*=*R*_1_*R*_2_,…,*R*_*k*_ such that: 

− *R*_1_ is a reaction having a *source* node (i.e. an input only node);

− for each *i*,*j*∈ [ 1,*k*], *i*≠*j*, *R*_*i*_ and *R*_*j*_ are different reactions;

− for each *i*∈ [ 1,*k*-1], some of the output nodes of *R*_*i*_ are input nodes of *R*_*i*+1_;

− the length *k* of the path *p* is maximal.

We denote by $R_{H}$ the set of all the reaction paths in the hypergraph *H*. It is obtained through an in-depth search algorithm iterating over the *source* nodes of *H*.

This step results in the sets $R_{H_{1}}$ and $R_{H_{2}}$, which are the reaction paths of *H*_1_ and *H*_2_, respectively.

#### Reaction path alignment

The second step establishes a first correspondence between *H*_1_ and *H*_2_ in terms of their sets of reaction paths $R_{H_{1}}$ and $R_{H_{2}}$. This is done by performing an all-against-all alignment of the paths in $R_{H_{1}}$ and $R_{H_{2}}$. More precisely, two reaction paths $p\in R_{H_{1}}$ and $p^{\prime }\in R_{H_{2}}$ are aligned using the classical Smith-Waterman sequence alignment algorithm [25], where the similarity between a reaction *R* in the path *p* and a reaction *R*^′^ in the path *p*^′^ is given by *SimReact*(*R*,*R*^′^). The score of the obtained sequence alignment is denoted by *scorePath*(*p*,*p*^′^).

#### Reaction path matching

The third step refines the correspondence between *H*_1_ and *H*_2_ by defining a matching $\sigma \subseteq R_{H1}\times R_{H2}$ that associates a path in $R_{H_{1}}$ with its ‘most similar’ path in $R_{H_{2}}$. This is done by defining a complete bipartite graph where the nodes are the reaction paths in $R_{H1}$ and $R_{H2}$ and the edge weight between two nodes (paths) *p* and *p*^′^ is the score *scorePath*(*p*,*p*^′^) of their sequence alignment obtained in the previous step. The matching *σ* is the result of the maximum weighted bipartite matching algorithm applied to the complete bipartite graph.

Recall that a matching *M* on a bipartite graph is a subset of edges such that no two edges in *M* share an endpoint. The cost of M is the sum of the cost of its edges. A matching is called a *maximum weight matching* if its cost is at least as great as the cost of any other matching.

Consider, for example, the KEGG pathway *Arginine and proline metabolism* for the organisms *Homo Sapiens* (hsa00330) and *Methanocaldococcus Jannaschii* (mja00330). Once they have been represented as hypergraphs, the matching between their reaction paths and the corresponding score can be computed, as shown in Table 1.

**Table 1:** Reaction paths and alignment

**Path alignment hsa00330-mja00330**	
Score: 0.412533333333	
R00669	-
R00667	R02282rev
R00667-183rev	R02282
Score: 0.835869605625	
R00669	R09107
R01398	-
R01954	R01954
R01086	R01086
R00566	R00566
R01157	R01157
R01920	R01920
R02869	R02869
Score: 1.0	
R00178	R00178
R01920	R01920
R02869	R02869
Score: 0.348801372167	
R00135	R00259-176
R01251	-
R00708	R02649
R00245rev	R03443
R00667rev	R02283
-	R00669-181
R00667	R02282rev
Score: 0.70616735275	
R00135	R00259
R01251	-
R00708	R02649
R00245rev	R03443
R00667rev	R02283
-	R00669
R01398	R01398
R01954	R01954
R01086	R01086
R00566	R00566
R01157	R01157
R01920	R01920
R02869	R02869
Score: 0.5	
R05051	-
R05052	R05052
Score: 0.2118750495	
R01991rev	R00253
R01989	-
Score:0.2715741285	
R00256	R03187rev
R00248rev	R03187
Score:0.3869154844	
-	R00259
-	R02649
R03313	R03443
R00667rev	R02283
R00667	R02282

#### Reaction matching

The fourth step translates the reaction path matching *σ* into a well-defined matching between reactions in *H*_1_ and reactions in *H*_2_. This is done by analyzing the alignments of all pairs of reaction paths (*p*,*p*^′^)∈*σ* and by building a corresponding *match-frequency matrixM* whose rows and columns represent the reactions (hyperedges) of *H*_1_ and *H*_2_, respectively. Each entry *m*_*i*,*j*_ of the matrix *M* counts the number of times that the reaction *R*_*i*_ in *H*_1_ is aligned to the reaction *R*_*j*_ in *H*_2_ in all pairs of reaction paths (*p*,*p*^′^)∈*σ*.

Suppose, for example, that reaction *R*_*i*_ appears in *k* reaction paths in $R_{H1}$ and that *R*_*i*_ is aligned to *R*_*j*_*k*^′^ times (with *k*^′^≤*k*) in the corresponding paths of $R_{H2}$ (through *σ*). In this case, the match-frequency matrix records the value *m*_*i*,*j*_=*k*^′^.

Once the matrix *M* has been determined, the best match between reactions is sought, taking care to associate each reaction in *H*_1_ with exactly one reaction in *H*_2_. This is done by employing, once again, the maximum weighted bipartite matching algorithm: given the frequency matrix *M* as input, it produces a matching *ρ*⊆*E*_1_×*E*_2_ as output, which provides the final reaction matching between *H*_1_ and *H*_2_.

#### Final score and hypergraph alignment

The fifth and last step of the algorithm determines the similarity score and the alignment of the two given hypergraphs. Intuitively, the similarity score considers all pairs of their ‘most similar’ reactions (determined by *ρ*) and sums the score of the ‘most similar’ paths they belong to (determined by *σ*), thus taking into account the topology of the two given pathways. Formally, the similarity score of *H*_1_ and *H*_2_ is defined as follows:

$$\mathrm{Score}(H_{1},H_{2})=\frac{\underset{(R,R^{\prime })\in \rho }{\sum }\mathrm{maxscorePath}(R,R^{\prime }))}{\max\{|E_{1}|,|E_{2}|\}}$$

where

$$\begin{array}{ll} \;\mathrm{maxscorePath}(R,R^{\prime }))=\;\max\{\mathrm{scorePath}(p,p^{\prime })\;|\;(p,p^{\prime })\in \sigma ,\;\\ \;R\in p,R^{\prime }\in p^{\prime },(R,R^{\prime })\in \rho \}.\; & \; \end{array}$$

The final alignment of *H*_1_ and *H*_2_ is defined in terms of their largest conserved substructure (sub-hypegraph). More precisely, the alignment of *H*_1_ and *H*_2_ is determined by using the reaction matching *ρ* to build a *relational graph* G as follows: 

− the nodes of G are the reactions in *H*_1_

− an edge (*R*_*i*_,*R*_*j*_), with *R*_*i*_, *R*_*j*_ reactions in *H*_1_, is introduced in G if and only if 

− some output nodes of *R*_*i*_ are input nodes of *R*_*j*_, i.e. they are connected hyperedges in *H*_1_, and

− some output nodes of *ρ*(*R*_*i*_) are input nodes of *ρ*(*R*_*j*_), i.e. their images through *ρ* are also connected hyperedges in *H*_2_.

Intuitively, the relational graph G expresses the connections between the reactions matched by *ρ*. The largest connected subgraph in the relational graph G corresponds to the largest conserved substructure (subpathway) between *H*_1_ and *H*_2_ through *ρ* and defines the final alignment of the two hypergraphs.

Let’s consider once again the hypergraphs corresponding to the KEGG pathway *Arginine and proline metabolism* for *H. Sapiens* (hsa00330) and *M. Jannaschii* (mja00330). The final alignment obtained by **MP-Align** is shown in Table 2. In this case, the largest conserved substructure (subpathway) contains the common reactions appearing in the *Urea Cycle* (highlighted in boldface).

**Table 2:** Final alignment hsa00330-mja00330

**hsa-mja alignment**	**<->**	**Enzyme**
**R01398**	<->	ec:2.1.3.3
**R01954**	<->	ec:6.3.4.5
R00566	<->	ec:4.1.1.19
R01920	<->	ec:2.5.1.16
R00178	<->	ec:4.1.1.50
R02869	<->	ec:2.5.1.16
R01157	<->	ec:3.5.3.11
**R01086**	<->	ec:4.3.2.1

Common reactions appearing in the *Urea Cycle* are highlighted in boldface.

#### Complexity and execution time

The complexity of the **MP-Align** algorithm is exponential in the size of the two input hypergraphs. This is already true in its first step, the Reaction path computation. Nevertheless, in our experience, **MP-Align** works fine on the hypergraphs representing metabolic pathways. To give an idea of the **MP-Align** efficiency, we report its execution times for the phylogeny recovery test illustrated in the next Section. It is a complex test that compares all the common pathways of eight selected organisms: there are 40 common pathways and there are 1440 pairwise comparisons and alignments to be performed; that is, **MP-Align** is executed 1440 times. We used a server with 16 processors at 2500 MHz and 24 GB of RAM memory. Since **MP-Align** is sequentially implemented, each pairwise comparison was performed by one processor. For this test, 30% of the pairwise comparisons and alignments were executed in 0.6 seconds at most; 60% were executed in 1.23 seconds at most; 90% were executed in 5.61 seconds at most and the 100% were executed in 4570.88 seconds at most. More precisely, only four pairwise comparisons and alignments were performed in more than one hour.

## Results and discussion

This section reports the tests performed with **MP-Align** to validate our similarity score and alignment algorithm. The statistical analysis was done using the **R**[26] basic package.

The first group of experiments employed cluster analysis methods to assess whether our similarity score and alignment algorithm could use metabolic information to provide organism classifications that are correct from the evolutionary point of view. The second group of experiments sought to validate the recognition and alignment of pairs of pathways that are known to contain functionally similar subunits but have different reaction sets and topologies.

### Data analysis

The first test of a similarity score between objects is typically cluster analysis, in which biological data objects are partitioned into groups such that the objects in each group share common traits.

#### First test on the Glycolysis pathway

The first test considered the *Glycolysis* pathway of all organisms in the KEGG database, which currently contains 1758 organisms: 52 Animals, 118 Archaea, 1491 Bacteria, 53 Fungi, 18 Plants and 51 Protists. We used **MP-Align** to compute the similarity score of all pairs of organisms and then converted the similarity score into the following distance measure:

(2) $$d(H_{1},H_{2})=\sqrt{2(1-\mathrm{Score}(H_{1},H_{2}))}$$

**Figure 3:**
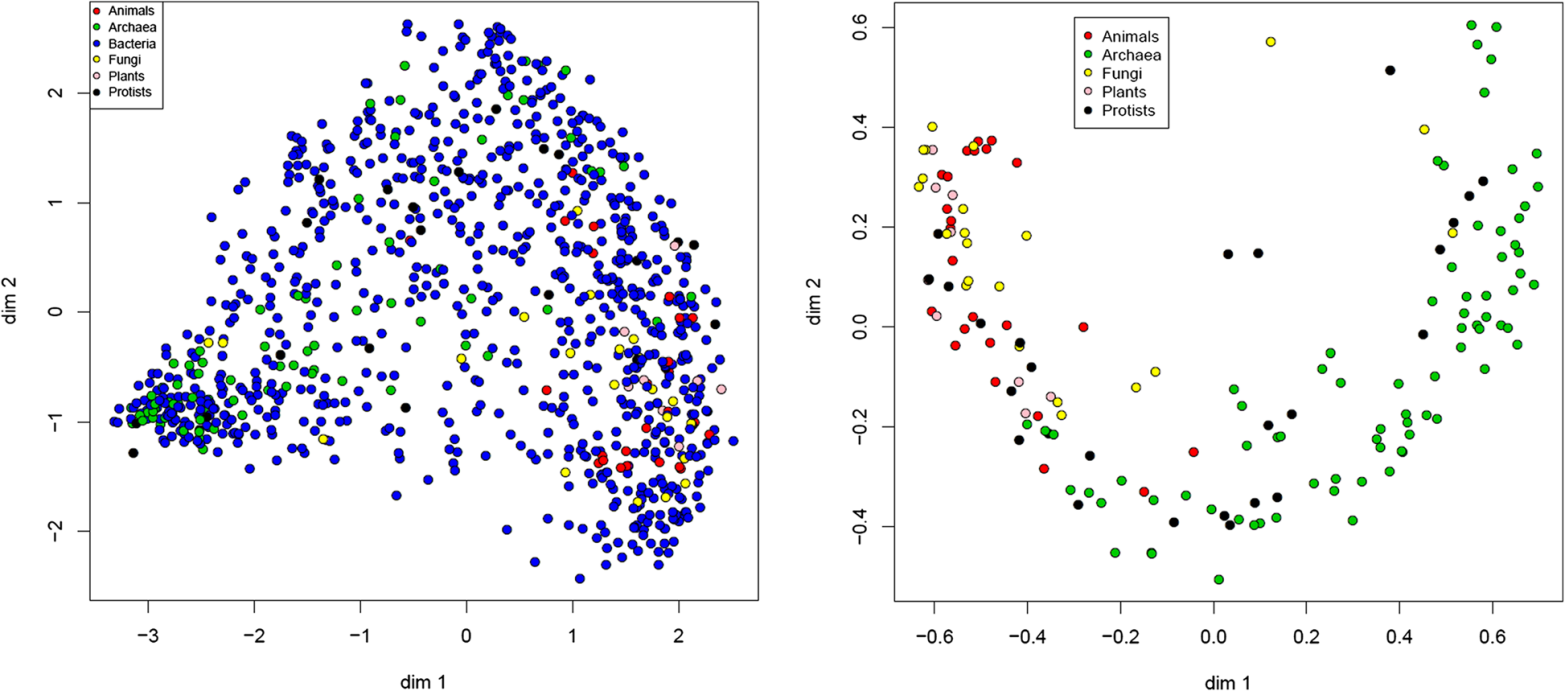
**Two-dimensional projections of the *Glycolysis *pathways of all organisms in the KEGG database (left) and all organisms up to Bacteria (right).** Red points correspond to Animals, green points to Archaea, yellow points to Fungi, pink points to Plants, black points to Protists and blue points to Bacteria. Note that in the projection on the left, Bacteria appears in the whole *Glycolysis* universe. By removing Bacteria, we can observe, on the right, that Protists are scattered throughout the whole space while Archaea are clearly separated from Animals, Plants and Fungi.

The results were visualized and analyzed using a classical multidimensional scaling (MDS) method. We represented the considered pathways as points in a two-dimensional space: the more distant the points in space, the less similar the corresponding pathways with respect to the considered distance. The results are shown on the left side of Figure 3. Note that Bacteria appears in the whole *Glycolysis* universe of the two-dimensional MDS. This could be due to the fact that there are considerably more Bacteria than other organisms, and a higher dimensional representation is required to discriminate between them and the other domains.

The test was repeated with all the previous domains except the Bacteria. Moreover, after noting that some of the KEGG *Glycolysis* pathways are identical for different organisms, we selected one representative from each group of organisms with an identical pathway. Table 3 shows the groups of organisms with identical *Glycolysis* pathways. Note that the various groups are homogeneous w.r.t. the classification into Bacteria, Archaea, Protists, Fungi, Plants and Animals, up to one group comprising Arthropods and Plants. We ended up with 160 different *Glycolysis* pathways. The results of this test are shown on the right side of Figure 3. Note that Protists are scattered throughout the whole space, while Archaea are clearly separated from Animals, Plants and Fungi.

**Table 3:** **Organisms sharing an identical *Glycolysis * pathway in the KEGG database**

**Equal** ***Glycolysis* pathways**	**Classification**
sce, kla, vpo, zro, dha, pic,	
pgu, lel, cal, ctp, cdu, clu,	Fungi
bfu, nfi, aor, afv, pcs, cpw, ure	
dan, der, dpe, dse, dwi, dya,	
dgr, dmo, dvi, aga, cqu, nvi,	Animals/Arthropods
gmx, bdi, smo, mbr	Plants
hsa, ptr, pon, mcc, mmu, rno, aml,	Animals/Vertebrates
bta, ecb, mdo, gga, acs, xtr, dre	
sso, sis, sia, sim, sid, siy, sin, sii	Archaea
ath, aly, pop, rcu, vvi, zma, ppp	Plants
lth, ncr, pan, mgr, fgr, afm, act	Fungi
mfe, mmq, mmx, mmz, mmd, mok	Archaea
mja, mvu, mfs, mae, mvn	Archaea
mth, mmg, msi, mel, mew	Archaea
ago, yli, lbc	Fungi
tml, cci, scm	Fungi
pfh, pbe, pkn	Protists
hla, htu, hxa	Archaea
pab, ton, tba	Archaea
dka, dmu, tag	Archaea
pcl, pyr, pog	Archaea
hhi, hbo	Archaea
cfa, mgp	Animals/Vertebrates
cin, dpo	Animals/Ascidians
cbr, bmy	Animals/Nematodes
olu, ota	Plants
ppa, cgr	Fungi
smp, pte	Fungi
cne, cnb	Fungi
ehi, edi	Protists
pfd, pyo	Protists
tan, tpv	Protists
mif, mig	Archaea
mac, mba	Archaea
mbu, mmh	Archaea
mhu, mem	Archaea
mpl, fpl	Archaea
hsl, hmu	Archaea
tac, tvo	Archaea
pfu, tko	Archaea
pyn, pya	Archaea

Each row-box shows the organisms sharing the same pathway and the corresponding classification.

#### Second test on the Glycolysis pathway

This test combined hierarchical clustering and pathway alignment. The idea was first to compare a set of pathways using our similarity score and produce a hierarchical clustering, and then to use our alignment algorithm to look for the largest conserved motifs in each cluster. The latter was done by computing the pairwise alignments of the pathways in each cluster (in a predetermined order) and by considering their common set of aligned reactions, that is, the intersection of their largest common motif. The overall goal was to explore whether the alignment technique could help in validating, or detecting the flaws of, the clustering results. Consider, for instance, two organisms having an identical pathway that forms a connected hypergraph. Now suppose that a reaction is removed from one of the pathways disconnecting its hypergraph. In this case, the similarity score considers the two organisms very close together, while their largest common motif reveals their structural difference. In fact, the comparison of two given pathways is based on their underlying sets of reactions and ignores their structure. A subsequent alignment phase includes structural information as well.

We focused on the *Glycolysis* pathway of Animals. In KEGG there are currently 53 distinct Animals having 25 distinct *Glycolysis* pathways. Table 3 shows the groups of organisms with an identical *Glycolysis* pathway in each row. Here as well, we took just one representative from each group of Animals. We performed the hierarchical clustering using Ward’s method [27] as well as the single, average and complete linkage methods to obtain a hierarchical clustering of the 25 pathways. All the methods form a distinguished cluster of Vertebrates, but do not allow for a fine grain distinction within the Invertebrates. We chose the dendrogram obtained by Ward’s method, because it better separates Vertebrates and Invertebrates.

**Figure 4:**
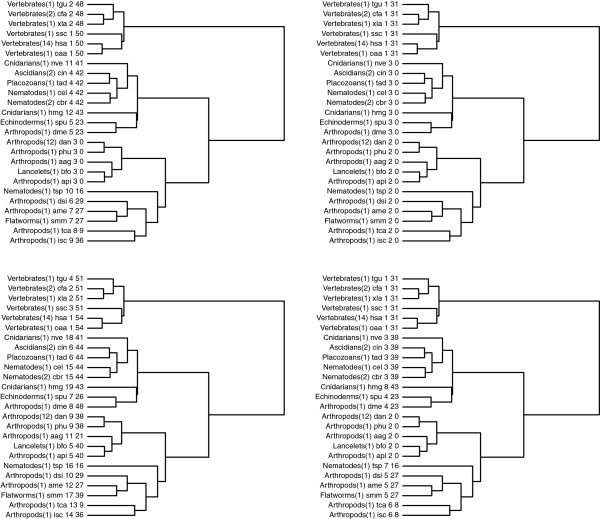
**Dendrograms of the hierarchical clustering with partitions into 3 (top left), 8 (top right), 12 (bottom left) and 19 (bottom right) clusters for the *Glycolysis *** **pathways of all Animals in the KEGG database.**

The dendrogram can be cut at different heights to obtain different partitions of the 25 pathways. We considered the cuts producing a total number of clusters ranging from 3 to 20, resulting in 18 different partitions. This allowed us to observe how the clusters evolve by incrementing their total number. For each partition, we looked for the conserved motifs in each cluster using the procedure described above, and we observed how the common motifs evolve as the number of clusters increases. In Figure 4 we show the most relevant partitions: we consider 3 clusters (top left dendrogram), 8 clusters (top right dendrogram), 12 clusters (bottom left dendrogram) and 19 clusters (bottom right dendrogram), respectively. Each leaf in the dendrograms represents a specific organism or the representative of a group sharing an identical *Glycolysis* pathway. The label of each leaf reports the classification of the organism, the number of represented organisms (within parenthesis), the organism name (in KEGG nomenclature), the cluster number, and the size of the common motif in the cluster (in terms of the number of reactions). For singleton clusters, the latter is just the number of reactions in the largest connected component of the organism itself.

One can note how the clusters evolve by incrementing their total number, and how the common motifs for each cluster become more and more significative. In particular, Vertebrates are separated from all other Animals from the very start, and their alignment confirms that they form a very cohesive cluster. In fact, in the top left and right dendrograms, the Vertebrates cluster has a common motif composed of 31 reactions. In the bottom left and right dendrograms this cluster is refined into two different clusters, with a common motif of size 48 and 50, respectively. In the top left dendrogram none of the other clusters share a common motif. This means that there are structural differences among the pathways in each cluster that could not be captured by the similarity score. In the top right dendrogram only cluster number 2 lacks a common motif. This remains true for cluster number 3 (composed of the same organisms) in the bottom left dendrogram. A closer look at the *Glycolysis* pathway of these organisms reveals that the *Aedes Aegypty* (aag) *Glycolysis* pathway is disconnected, so it can hardly share a common motif with the other organisms. When considering the 19 final clusters in the bottom right dendrogram, *Aedes Aegypty* forms a singleton cluster, and the other organisms are divided into two clusters, both having quite significative conserved motifs. The structural difference of the *Aedes Aegypty Glycolysis* pathway, invisible to the similarity score, could be revealed by the alignment phase.

Other organisms whose *Glycolysis* pathway is disconnected are *Tribolium Castaneum* (tca), *Apis Mellifera* (ame) and *Trichinella Spiralis* (tsp). Notice in the dendrograms that, as soon as these organisms are isolated (by increasing the number of clusters), the conserved motifs in the newly formed clusters can evolve.

**Figure 5:**
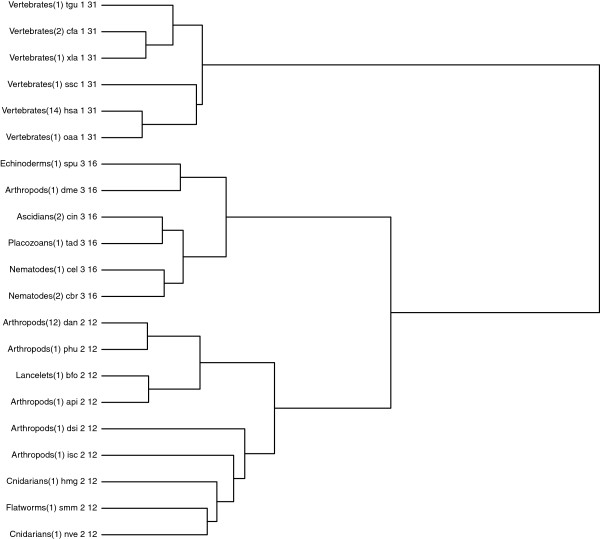
**Dendrogram of the hierarchical clustering with partition into 3 clusters for the *Glycolysis *** **pathways of all Animals in the KEGG database having a connected *Glycolysis *** **pathway.**

For the sake of completeness we repeated the same test without including the organisms with a disconnected *Glycolysis* pathway. Figure 5 shows the hierarchical clustering obtained by Ward’s method and exhibits a partition into 3 clusters. By comparing the resulting dendrogram with the top left dendrogram in Figure 4 one can notice that all clusters now share a quite significant motif, which is to say, the absence of the outlier organisms allow them to be more cohesive.

#### Recovering phylogenies

One of the questions that arises when comparing metabolic pathways is whether it is possible to reconstruct robust phylogenetic trees from non-genomic data such as metabolic pathways. In [7] the authors argue that this is indeed the case, by presenting a method to assess the structural similarity of metabolic pathways for several organisms. On the basis of their similarity measure, the authors were able to reconstruct phylogenies similar to the NCBI reference taxonomy [28]. One of their experiments considered all the common metabolic pathways (taken from KEGG) of the following eight organisms: *A. Fulgidus* (afu), *C. Perfringens* (cpe), *H. Influenzae* (hin), *L. Innocua* (lin), *M. Jannaschii* (mja), *M. Musculus* (mmu), *N. Meningitidis* (nme) and *R. Norvegicus* (rno). They belong to Bacteria (cpe, hin, lin, nme), Archaea (mja, afu) and Animals (mmu, rno).

We repeated the same experiment using our similarity score. We performed the pairwise comparison of all organisms for each common pathway and combined the obtained scores as follows.

For any pair of organisms with *k* common pathways, we used the average score

$$\text{AverageScore}(O_{1},O_{2})=\frac{\overset{k}{\underset{i=1}{\sum }}\text{Score}(H_{1,i},H_{2,i})}{k}$$

and the following distance measure

$$d(O_{1},O_{2})=\sqrt{2(1-\mathrm{AverageScore}(O_{1},O_{2}))}.$$

**Figure 6:**
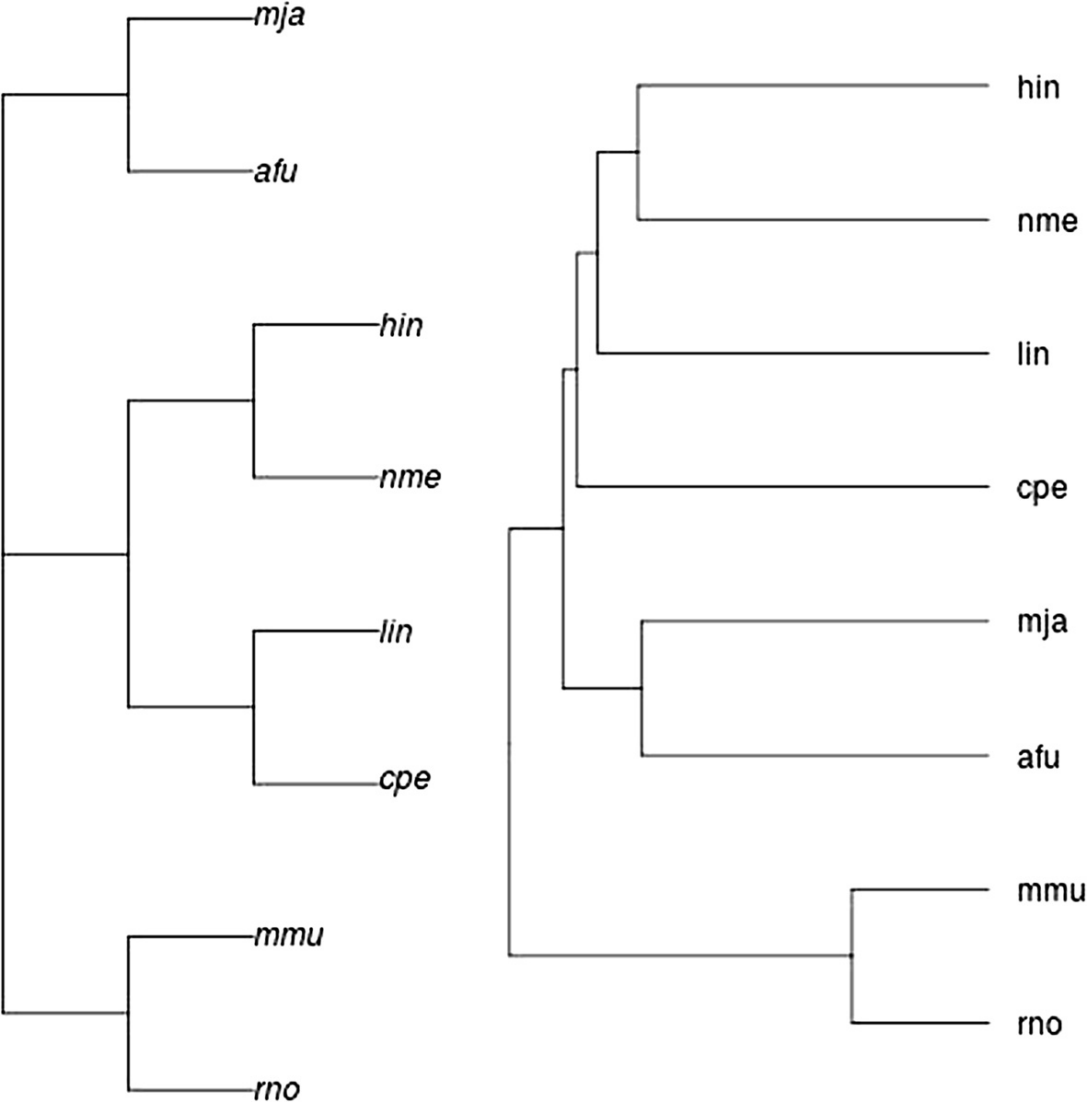
NCBI taxonomy of the eight organisms considered (left) and phylogenetic reconstruction obtained by MP-Align using the average score (right).

The average score is suitable in this case because it makes it possible to capture comprehensive information from the comparison among all common pathways of the given organisms. Once all the distance measures between organisms were obtained, we made a hierarchical clustering using the single, average and complete linkage methods. The three methods produced exactly the same clustering, thereby confirming the robustness of the average score employed. The result is reported on the right of Figure 6: our phylogenetic tree coincides with the one obtained in [7], and it is very close to the NCBI reference taxonomy of the same organisms, shown on the left of Figure 6. More precisely, if, for instance, we consider the Robinson-Foulds distance on phylogenetic trees [29], it is evident that the NCBI taxonomy tree shares four of its five clusters with the phylogenetic tree derived by using **MP-Align**. The only cluster that is not present in the phylogenetic tree is {*cpe*, *lin*}.

**Figure 7:**
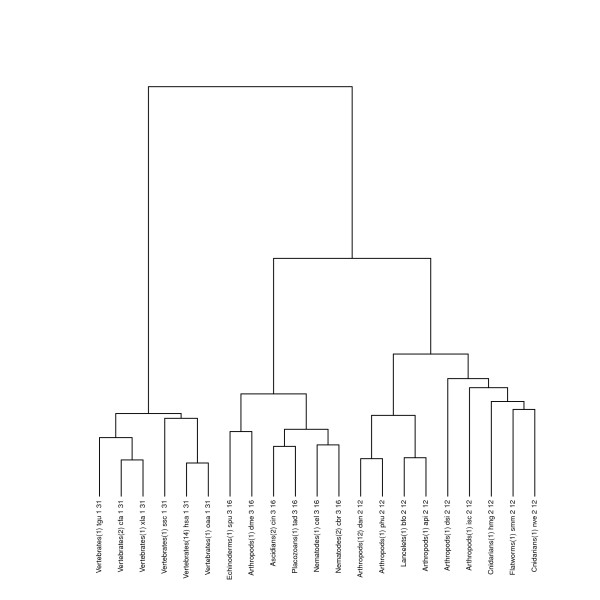
**Phylogenetic reconstruction obtained by MP-Align for the *Glycolysis *** **pathway (top left) and for randomly chosen subsets of the common pathways of the selected organisms: 10 pathways (top right), 20 pathways (bottom left) and 30 pathways (bottom right).**

We repeated the test considering just one pathway, the Glycolysis pathway, and also considering randomly chosen subsets of 10, 20 and 30 pathways. The resulting phylogenetic trees are shown in Figure 7: they do not recover exactly the phylogeny of the original test, but they all distinguish Bacteria, Archaea and Animals, and in this sense they confirm the validity of the adopted average score and the robustness of the obtained phylogeny. Actually, the phylogenetic tree resulting from the 30 randomly chosen pathways perfectly characterizes the Bacteria into two distinct clusters, {*cpe,lin*} and {*hin, nme*}, as in the NCBI taxonomy.

Therefore, we can conclude that the similarity score provided by **MP-Align** can reconstruct robust phylogenies that are meaningful and very close to the NCBI reference taxonomy.

### Metabolic pathway alignment

Several tests were performed to evaluate our alignment tool, some of which were taken from [30]. As explained in [30], an example in favor of the so-called *patchwork evolution model* is the *Urea Cycle*, which, in terrestrial animals, clearly evolved by adding a new enzyme, *Arginase*, to a set of four enzymes previously involved in the biosynthesis of *Arginine*[31]. Therefore, we considered the KEGG pathway *Arginine and proline metabolism* for *Homo Sapiens* (hsa), *Anolis Carolinensis* (acs), and *M. Jannaschii* (mja) and performed their alignment using **MP-Align**. Since *M. Jannaschii* belongs to the Archaea domain, the *Arginase* enzyme is not present in its pathway and urea is not synthesized. Instead, the reptile *A. Carolinensis* and the mammal *H. Sapiens* share part of the *Urea Cycle*. As a result, we learned that **MP-Align** can recognize the identical parts of the *Urea Cycle* when comparing *H. Sapiens* and *A. Carolinensis* and finds a mismatch when comparing *H. Sapiens* and *M. Jannaschii*. Table 1 shows the reaction path alignment obtained by **MP-Align** when considering the *Arginine and proline metabolism* for *H. Sapiens* and *M. Jannaschii*. Note that the highest score is about 0.836, which corresponds to the reaction path alignment starting at *N-Acetyl-L-citrulline* for *M. Jannaschii* and at *N-Acetylornithine* for *H. Sapiens* and both ending at *Spermine*, where the *β-Alanine metabolism* or the *Glutathione metabolism* is reached. Thus, in its first step, **MP-Align** is able to recognize and align the longest path that both organisms share. Moreover, Table 4 and Table 5 show the reaction matchings obtained by **MP-Align** when reconsidering the *Arginine and proline metabolism* for *H. Sapiens*, *A. Carolinensis* and *M. Jannaschii*. The reactions that appear in the *Urea Cycle* are shown in boldface. The reaction catalyzed by the *Arginase* enzyme, *R00551* in KEGG nomenclature, only appears when considering *H. Sapiens* and *A. Carolinensis* (see Table 5). Instead, when considering *H. Sapiens* and *M. Jannaschii*, *R00551* is not aligned (see Table 4): the reactions in boldface are in the upper part of the *Urea Cycle* but the cycle is incomplete. Table 6 shows the final alignment between *H. Sapiens* and *A. Carolinensis*: the enzymes catalyzing the reactions are listed for easy recognition in the KEGG pathway map. It is evident that all the reactions in the *Urea Cycle* (in boldface) are conserved, and the whole cycle is correctly aligned. Table 2 shows the final alignment between *H. Sapiens* and *M. Jannaschii*: note that reaction *R*00551 is not aligned and, consequently, the *Urea Cycle* is not a common conserved subpathway.

**Table 4:** Reaction matching hsa00330-mja00330

**hsa Reactions**	**<->**	**mja Reactions**
R01253	<->	R03187
R01251	<->	R02649
R00670	<->	R02282
R00667	<->	R02282rev
**R01954**	<->	**R01954**
R00708	<->	R03443
R02894	<->	R00253
R02869	<->	R02869
R00245rev	<->	R02283
R01157	<->	R01157
**R01086**	<->	**R01086**
R00565rev	<->	R00259
**R01398**	<->	**R01398**
R00667rev	<->	R00669
R00669	<->	R09107
R00178	<->	R00178
R01920	<->	R01920
R00135	<->	R03187rev
R05051	<->	R05052
R00566	<->	R00566

Reactions appearing in the *Urea Cycle* are highlighted in boldface.

**Table 5:** Reaction matching acs00330-hsa00330

**acs Reactions**	**<->**	**hsa Reactions**
R01252	<->	R01252
R00557rev	<->	R01954
R01253	<->	R01253
R01154	<->	R01154
R00565	<->	R00565
R00248rev	<->	R00245
R04025	<->	R04025
R00239	<->	R00239
R01992	<->	R01992
R03313	<->	R03313
R05050	<->	R05050
R01251	<->	R01251
R05052	<->	R05052
R00670	<->	R00670
R00667	<->	R00667
**R00551**	<->	**R00551**
R02894	<->	R02894
R04221	<->	R04221
R00248	<->	R00248
R02869	<->	R02869
R00256	<->	R00256
R00149	<->	R00149
R01991rev	<->	R01991rev
R02869	<->	R02869
R03293	<->	R03293
R02549	<->	R02549
R00558	<->	R00558
R05051	<->	R05051
R00565	<->	R00565rev
R00253	<->	R00253
R01991	<->	R01991
**R01398**	<->	**R01398**
R00667	<->	R00667
R00669	<->	R00669
R01992	<->	R01992
R00178	<->	R00178
R01920	<->	R01920
R00135	<->	R00135
**R00557**	<->	**R00557**
R01881	<->	R01881
R01251rev	<->	R01251rev
R03295	<->	R03295
R00566	<->	R00566
R01989	<->	R01989
R00111	<->	R00111
R01883	<->	R01883

Reactions appearing in the *Urea Cycle* are highlighted in boldface.

**Table 6:** Final alignment hsa00330-acs00330

**hsa-acs alignment**	**<->**	**Enzyme**
R00557rev	<->	ec:1.14.13.39
**R01398**	<->	ec:2.1.3.3
**R00557**	<->	ec:1.14.13.19
R00670	<->	ec:4.1.1.17
R00670	<->	ec:4.1.1.17
R00248	<->	ec:1.4.1.3
R00256	<->	ec:3.5.1.38
R03313	<->	ec:1.2.1.41
R00565rev	<->	ec:2.1.4.1
**R00551**	<->	ec:3.5.3.1
R00566	<->	ec:4.1.1.19
R00558	<->	ec:1.14.13.39
R00565	<->	ec:2.1.4.1
R04025	<->	ec:1.4.3.4
R00178	<->	ec:4.1.1.50
R02869	<->	ec:2.5.1.16
R02869	<->	ec:2.5.1.22
R00239	<->	ec:2.7.2.17
R00248rev	<->	ec:1.4.1.3
R01883	<->	ec:2.1.1.2
R00111	<->	ec:1.14.13.39
R00667rev	<->	ec:2.6.1.13
R00669	<->	ec:3.5.1.14
R01920	<->	ec:2.5.1.16
R01154	<->	ec:2.3.1.57
R00149	<->	ec:6.3.4.16
R01881	<->	ec:2.7.3.2
R00253	<->	ec:6.3.1.2
R05050	<->	ec:1.2.1.3

Reactions appearing in the *Urea Cycle* are highlighted in boldface.

To complete the validation of **MP-Align**, an attempt was made to compare it to the SubMAP alignment tool [18]^a^. This comparison was limited by the fact that the SubMAP utility required to translate KEGG pathways into the SubMAP input formalism is no longer available. Our analysis had to rely on previously translated pathways, namely the *Arginine and proline metabolism pathway* for *H. Sapiens*, *S. Cerevisiae* and *C. Elegans*.

Focusing once again on the *Urea Cycle* of the selected organisms, we observed that *H. Sapiens* and *S. Cerevisiae* share the same *Urea Cycle*, while urea is not synthesized in *C. Elegans*. We performed the alignment between *H. Sapiens* and *S. Cerevisiae* and *H. Sapiens* and *C. Elegans* using both **MP-Align** and SubMAP. As shown in Table 7, both tools were able to correctly match the reactions involved in the *Urea Cycle* (highlighted in boldface) in *H. Sapiens* and *S. Cerevisiae*. However, when considering the complete reaction matching done by both tools, it is clear that they perform quite differently: the **MP-Align** reaction matching appears to be more thorough.

**Table 7:** hsa00330-sce00330: reaction matching obtained by SubMAP and MP-Align

**SubMAP - reaction matching**			**MPAlign - reaction matching**		
**hsa00330**	***<->***	**sce00330**	**hsa00330**	***<->***	**sce00330**
R00243	<->	R00243	R01992	<->	R00774#rev
R00245	<->	R00245	R00239	<->	R00239
R00248	<->	R00248	R00670	<->	R00248
R00551	<->	R00551	**R01086**	<->	**R01086**
R00667	<->	R00667	R01251	<->	R01251
R00707	<->	R00707	R05052	<->	R05052
R00708	<->	R00708	R00565#rev	<->	R02283
**R01086**	<->	**R01086**	R00667	<->	R00667
R01248	<->	R01248	**R01954**	<->	**R01954**
R01251	<->	R01251	R00551	<->	R00551
R01253	<->	R01253	R02894	<->	R02922
**R01398**	<->	**R01398**	R00248	<->	R00243
**R01954**	<->	**R01954**	R00708	<->	R00708
R03293	<->	R03291	R02869	<->	R02869
R03295	<->	R03293	R00253	<->	R00253
R03646	<->	R03646	R00256	<->	R04445
R03661	<->	R03661	R02869	<->	R02869
R04444	<->	R04444	R00248#rev	<->	R00248#rev
R04445	<->	R04445	R01157	<->	R00670
R05051	<->	R05051	R02549	<->	R00774
R05052	<->	R05052	R00245#rev	<->	R00259
			R05051	<->	R05051
			R05051	<->	R05051
			R00667#rev	<->	R00667#rev
			R03313	<->	R03313
			R04221	<->	R03293
			R04025	<->	R03443
			R00669	<->	R00243#rev
			R00178	<->	R00178
			**R01398**	<->	**R01398**
			R01920	<->	R01920
			R00135	<->	R02649
			R00245	<->	R00245
			R00557#rev	<->	R02282#rev
			R01881	<->	R00005
			R00557	<->	R00245#rev
			R01251#rev	<->	R01251#rev
			R00566	<->	R02282
			R01991#rev	<->	R05050
			R01989	<->	R03180
			R01253	<->	R01253

Reactions appearing in the *Urea Cycle* are highlighted in boldface.

Concerning the alignment between *H. Sapiens* and *C. Elegans* the two tools performs differently. As evident in Table 8, SubMAP matches reaction *R00565* (catalyzed by enzyme 2.1.4.1) with reaction *R00554* (catalyzed by enzyme 2.7.3.3), although the two reactions belong to different parts of the pathways. The wrong match is highlighted in boldface. In the matching provided by **MP-Align**, however, reaction *R00565* is not matched, so it is not included in the final alignment of the two pathways.

**Table 8:** hsa00330-cel00330: reaction matching obtained by SubMAP and MP-Align

**SubMap - reaction matching**			**MPAlign - reaction matching**		
**hsa00330**	**<->**	**cel00330**	**hsa00330**	**<->**	**cel00330**
R00243	<->	R00243	R01991#rev	<->	R04445
R00245	<->	R00245	R00239	<->	R00239
R00248	<->	R00248	R05052	<->	R05052
**R00565**	<->	**R00554**	R01251	<->	R01251-
R00667	<->	R00667	R00670	<->	R00670
R00707	<->	R00707	R02894	<->	R02894
R00708	<->	R00708	R00248-	<->	R00248
R01248	<->	R01248	R00708	<->	R00708
R01251	<->	R01251	R00256	<->	R00256
R01252	<->	R01252	R02869	<->	R02869
R01253	<->	R01253	R00248#rev	<->	R00248#rev
R02894	<->	R02894	R00557	<->	R00554
R03293	<->	R03291	R01989	<->	R03293
R03295	<->	R03293	R00245#rev	<->	R00245#rev
R03646	<->	R03646	R05051	<->	R05051
R03661	<->	R03661	R05051	<->	R05051
R04221	<->	R04221	R00253	<->	R00253
R04444	<->	R04444	R00667#rev	<->	R00667#rev
R04445	<->	R04445	R03313	<->	R03313
R05051	<->	R05051	R04221	<->	R04221
R05052	<->	R05052	R00565#rev	<->	R00669
			R00178	<->	R00178
			R01920	<->	R01920
			R00245	<->	R00245
			R00667	<->	R00667
			R00669	<->	R05050
			R00135	<->	R01251#rev
			R01253	<->	R01253

A wrong match is highlighted in boldface.

This test revealed a difference between the two tools, which became evident when reporting their matchings back to the corresponding KEGG maps. The matchings computed by SubMAP allow the alignment of individual reactions, or small groups of reactions, without considering the topology of the whole pathway. **MP-Align** takes the entire network topology into account in the final alignment, thereby identifying the largest connected subpathway.

## Conclusions

This paper presents a new methodology and tool for the pairwise comparison and alignment of metabolic pathways. The methodology is based on a hypergraph representation of metabolic pathways and defines a reaction similarity score based on enzyme and compound similarities. The proposed alignment technique uses the adopted reaction similarity score as well as the pathway topology to identify the largest conserved subpathway between the two given pathways.

We used our tool **MP-Align** to perform several tests to validate the proposed similarity score and alignment algorithm. The first was a comparative analysis test that showed that our approach allows for discriminating between different domains. The second was a phylogenetic reconstruction test that showed that, by considering all the common pathways of eight specific organisms, our approach makes it possible to recover a robust phylogeny that is very close to the NCBI reference taxonomy of those organisms. The last was an alignment test that showed that our alignment algorithm correctly identifies subpathways sharing a common biological function.

Finally, we performed a comparison between **MP-Align** and the SubMAP alignment tool [18]. The two tools seem to have been designed for different purposes: SubMAP looks for small conserved substructures while **MP-Align** identifies the largest conserved subpathway.

## Endnote

^a^ SubMAP allows the matching between reactions to be either one-to-one (one reaction is matched to exactly one reaction) or one-to-many (one reaction can be matched to many – maximum five – reactions). We performed our tests using the one-to-one alternative.

## Competing interests

The authors declare that they have no competing interests.

## Authors’ contributions

MT and DS implemented the algorithms. RA designed and analyze the tests. ML and MS conceived the methods and wrote the paper. All authors read and approved the final manuscript.
